# Transcriptome Profiling Reveals Differences Between Rainbow Trout Eggs with High and Low Potential for Gynogenesis

**DOI:** 10.3390/genes16070803

**Published:** 2025-07-08

**Authors:** Konrad Ocalewicz, Artur Gurgul, Stefan Dobosz, Igor Jasielczuk, Tomasz Szmatoła, Ewelina Semik-Gurgul, Mirosław Kucharski, Rafał Rożyński

**Affiliations:** 1 Department of Marine Biology and Biotechnology, Faculty of Oceanography and Geography, University of Gdańsk, M. Piłsudskiego 46 Av, 81-378 Gdynia, Poland; r.rozynski.874@studms.ug.edu.pl; 2 Center for Experimental and Innovative Medicine, University of Agriculture in Kraków, Redzina 1C, 30-248 Krakow, Poland; artur.gurgul@urk.edu.pl (A.G.); igor.jasielczuk@urk.edu.pl (I.J.); tomasz.szmatola@urk.edu.pl (T.S.); 3 Department of Salmonid Research, Inland Fisheries Institute in Olsztyn, Rutki, 83-330 Żukowo, Poland; dobosz@infish.com.pl; 4 Department of Animal Molecular Biology, National Research Institute of Animal Production, Krakowska 1, 32-083 Balice, Poland; ewelina.semik@iz.edu.pl; 5 Department of Animal Physiology and Endocrinology, University of Agriculture in Kraków, Mickiewicza 24/28, 30-059 Krakow, Poland; miroslaw.kucharski@urk.edu.pl

**Keywords:** doubled haploids, egg quality, maternal RNA, reproduction, RNA-seq, salmonids

## Abstract

Background/Objectives: Fish eggs activated with UV-irradiated spermatozoa and exposed to the High Hydrostatic Pressure (HHP) shock to inhibit first cell cleavage develop as gynogenetic Doubled Haploids (DHs) that are fully homozygous individuals. Due to the expression of the recessive genes and side effects of the gamete treatment, survival of fish DHs is rather low, and most of the mitotic gynogenotes die before hatching. Nevertheless, as maternal gene products provided during oogenesis control the initial steps of embryonic development in fish, a maternal effect on the survival of gynogenotes needs to be also considered to affect efficiency of gynogenesis. Thus, the objective of this research was to apply an RNA-seq approach to discriminate transcriptional differences between rainbow trout (*Oncorhynchus mykiss*) eggs with varied abilities to develop after gynogenetic activation. Methods: Gynogenetic development of rainbow trout was induced in eggs originated from eight females. Maternal RNA was isolated and sequenced using RNA-Seq approach. Survival rates of gynogenotes and transcriptome profiles of eggs from different females were compared. Results: RNA-seq analysis revealed substantial transcriptional differences between eggs originated from different females, and a significant correlation between the ability of the eggs for gynogenesis and their transcriptomic profiles was observed. Genes whose expression was altered in eggs with the increased survival of DHs were mostly associated (GO BP) with the following biological processes: development, cell differentiation, cell migration and protein transport. Some of the genes are involved in the oocyte maturation (*RASL11b*), apoptosis (*CASPASE 6*, *PGAM5*) and early embryogenesis, including maternal to zygotic transition (*GATA2*). Conclusions: Inter-individual variation of the transcription of maternal genes correlated with the competence of eggs for gynogenesis suggest that at least part of the mortality of the rainbow trout DHs appear before activation of zygotic genome and expression of the lethal recessive traits.

## 1. Introduction

The transcriptome is a functional component of the genome, and it is composed of a set of RNA transcripts, including both protein-coding and non-protein-coding RNAs expressed in a single cell or a population of cells [1]. In turn, transcriptomics refers to a comprehensive study of the complete set of transcripts for a specific cell, tissue, or organism at a particular developmental stage or physiological state. Transcriptomics is utilized in many biological studies to investigate diseases at the molecular level, explore gene functions, provide biomarkers of tissue and organism states, and assess the responses of organisms to environmental conditions, among other applications. NGS-based RNA-Seq technology provides an efficient and precise method for transcriptome profiling that measures the expression of numerous genes in cell populations, tissues or organisms in a single assay, making this approach advantageous over microarray analysis [2].

The increasing availability of RNA-Seq has enabled transcriptome profiling not only for humans and model organisms but also for wild plant and animal species, as well as those utilized in agriculture, animal breeding, and aquaculture. In aquaculture, transcriptome profiling can be applied to identify candidate genes related to development, sexual dimorphism, reproduction, growth, disease resistance, and immunology in cultivated plant and animal species [3]. Special attention has been given to the transcriptomic profiles of fish eggs, which contain exclusively maternal RNA deposited in the egg cytoplasm during oogenesis. It has been observed that fish farming conditions affect the expression of maternal genes [4], and profiles of gene expression differ in eggs at various stages of oogenesis [5]. The quantity of maternal RNA has also been examined to evaluate the developmental competence of fish eggs; in some species, transcriptomic differences between low- and high-quality eggs have been observed [4,6,7,8,9]. In this context, transcriptome profiling has been applied to identify differences in maternal gene expression between rainbow trout eggs exhibiting varied competence for mito-gynogenesis. Induced gynogenesis is a form of parthenogenesis in which fish eggs are activated by UV-irradiated spermatozoa, allowing embryonic development to proceed without the contribution of paternal chromosomes. Exposing gynogenetically activated eggs to thermal shock or high hydrostatic pressure (HHP) enables the diploidization of genetic material in gynogenetic haploid zygotes. The shock applied to eggs shortly after activation with irradiated spermatozoa (meiotic gynogenesis) leads to the abortion of the second polar body extrusion and the generation of heterozygous (meio)gynogenetic individuals. Conversely, physical shock implemented around the prophase of the first mitosis prevents the first cell cleavage (mitotic gynogenesis) and results in the generation of fully homozygous gynogenetic Doubled Haploids (DHs) [10]. DHs have been used in various aquaculture and model fish species to study the phenotypic consequences of recessive alleles, during selective breeding programs, and to generate isogenic and clonal lines [11,12]. The homozygosity of DHs simplifies detailed linkage analyses, enabling the identification of chromosome regions associated with quantitative trait loci (QTL) and significantly improving the de novo assembly of genomes sequenced using next-generation sequencing (NGS) approaches [13]. However, the use of mitotic gynogenesis in aquaculture is limited by the reduced survival rates of DHs. Few (if any) DH gynogenetic females survive up to sexual maturation, which impedes use of such individuals in the breeding programs and generation of clonal lines [14]. The high mortality of DHs may be explained by the expression of deleterious recessive alleles, alterations in the egg organelles caused by physical shock, and egg quality, which is the ability of eggs to be fertilized and develop into normal embryos.

In several species, significant variation in the efficiency of gynogenesis induced in eggs from different females has been observed. Such inter-clutch differences in the survival of gynogenotes may reflect inter-individual differences in the expression profiles of maternal genes. In our pilot experiment, eggs from one of four rainbow trout (*Oncorhynchus mykiss*) females demonstrated increased ability to develop after gynogenetic activation exhibited a different pattern of maternal gene expression [15]. These preliminary results raised the question of whether eggs from different females with enhanced competence for gynogenesis show similar transcriptomic profiles. To investigate this, we induced gynogenesis in eggs provided by eight rainbow trout females. Increased survival of gynogenotes was observed in eggs from four of these females. RNA-Seq analysis enabled the identification of several genes whose transcription levels were similar in eggs demonstrating increased developmental ability after activation with UV-irradiated sperm, and which differed significantly from those observed in eggs with reduced competence for gynogenesis.

## 2. Material and Methods

### 2.1. Fish Stock Origin and Maintenance

Rainbow trout and European grayling (*Thymallus thymallus*) gamete donors came from broodstocks kept at the Department of Salmonid Research, Inland Fisheries Institute in Olsztyn, Rutki, Poland. Eggs from eight 5-year-old rainbow trout females (F_1–8_), whose length (mm) and weight (g) varied from 540 to 655 and from 2440 to 4052, respectively (Supplementary Table S1), were collected and kept separately in plastic bowls at 10 °C pending further procedures. Semen from one 3-year-old rainbow trout (540 mm and 2288 g) and three 3-year-old grayling males, whose length and weight ranged from 310 to 400 mm and 242 and 552 g, respectively (Supplementary Table S1), was collected into separate plastic containers. The motility of the spermatozoa from each male was confirmed microscopically. The semen was then stored at +4 °C until further use.

### 2.2. Induction of Mitotic Gynogenesis

Mitotic gynogenesis in the rainbow trout was performed using standard conditions that have been established for the production of gynogenetic rainbow trout using European grayling sperm [14]. Semen from the three grayling males was mixed and diluted at a ratio of 1:40 in the rainbow trout seminal plasma. A total volume of 15.375 mL of the diluted semen was transferred to a 60 mL glass beaker, placed onto a magnetic stirrer (1400 G) 20 cm under the UV-C lamp (30 W) and irradiated (2075 μW/cm^2^) for 8 min [14,16]. Eight groups of gynogenotes (G_1–8_) were produced by the separate activation of eggs (n = ca. 4600) originating from each of eight females with the UV-irradiated grayling spermatozoa in the presence of the sperm-activating medium (154 mM NaCl, 20 mM Tris, 30 mM glycine, 1 mM CaCl_2_, pH 9.0) [17]. Five minutes after activation, eggs were rinsed with hatchery water. To duplicate haploid sets of the maternal chromosomes, eggs activated with the UV-irradiated spermatozoa were exposed to the high hydrostatic pressure (HHP) shock (9500 psi/3 min) exactly 350 min after insemination, using TRC-APV electric/hydraulic device (TRC Hydraulics Inc. Dieppe, Canada). The above conditions of HHP shock were proven to efficiently restore diploidy in the gynogenetically activated rainbow trout eggs and generate homozygous rainbow trout DHs [18]. Between activation and exposition for the HHP shock, eggs were incubated in a water bath at 10 °C. To establish control groups (C_1–8_), a portion of eggs (n = ca. 350) from each of the eight females were fertilized with the non-irradiated rainbow trout sperm. HHP shock was not applied to eggs from the control groups. Eggs from all experimental groups (G and C) were incubated in three separate replicates at 6–8 °C, using tray vertical incubators.

The survival rates in each experimental group at the eyed stage and at the swim-up stage were analyzed using Statistica software v.13.0 (StatSoft, Tulsa, OK, USA). Data distribution normality and homogeneity of variance were tested by Shapiro–Wilk and Levene’s test, respectively. ANOVA’s HSD Tukey test and Kruskal–Wallis’ test were used to determine significant differences in the survival rates of rainbow trout offspring in the experiment. Significance was established at the *p* < 0.05 level.

### 2.3. RNA Sequencing

Eggs activated with UV irradiated (G_1–8_) and non-irradiated (C_1–8_) sperm were collected immediately after treatment and preserved in RNAlater solution (Sigma Aldrich, Burlington, MA, USA), incubated overnight in a refrigerator (4 °C), and finally stored at −80 °C until RNA extraction and purification. Up to six eggs from each female were used for the transcriptomic analysis. A sample of two to three eggs, depending on their size, were mixed and used at once for the RNA isolation using a modified TRIzol (Thermo Scientific, Waltham, MA, USA) procedure [19] established at the Igor Babiak laboratory, Nord University, Bodø, Norway. The quality of the obtained RNA was evaluated using TapeStation4150 (Agilent Technologies, Santa Clara, CA, USA) and RNA ScreenTape Assay allowing RIN (RNA Integrity Number) evaluation. Good quality isolates (RIN > 8) were quantified using Qubit™ RNA Assay Kits (Thermo Scientific, Waltham, MA, USA), and 250 ng of a total RNA was used for library preparation with Illumina Stranded mRNA Prep kit (Illumina, San Diego, CA, USA) and standard protocol. The final libraries were controlled for quality using D1000 Agilent ScreenTapes and TapeStation4150 (Agilent Technologies, Santa Clara, CA, USA) system and quantified using Qubit™ dsDNA BR Assay Kit (Thermo Scientific, Waltham, MA, USA). An equimolar pool of libraries was finally commercially sequenced on NovaSeq6000 System (Illumina, San Diego, CA, USA) in a paired-end (PE) read of 2 × 150 bp to obtain a high sequencing depth (i.e., minimum of 75 million (M) PE reads per sample). Eggs from female no. 5 used for gynogenetic (G) and normal (C) activation were excluded from the analysis because of the poor sequencing library quality and insufficient amount of obtained reads. The raw sequencing reads were deposited in Sequencing Reads Archive (SRA) database under accession number SRP422754.

### 2.4. Data Analysis

Raw sequencing reads were checked for quality with the use of FastQC (v.0.11.9) software [20]. Then, reads were trimmed/filtered using Flexbar (v.3.5.0) software [21] to remove read ends with pored quality < 20, reads shorter than 35 bp and adapter sequences. Cleaned reads were mapped against rainbow trout OmykA_1.1 genome with the use of STAR aligner (v.2.7.5c) software [22]. HTSeq-count [23] (Anders et al., 2015) software was used to count the mapped reads into the respective Ensembl GTF annotation file (OmykA_1.1 version 108) features. After that, DESeq2 (v.3.16) software [24] was used for data normalization.

The DeSeq2 normalized read counts (representing separate genes expression) were used for correlational analysis with the survival rates of embryos developing in eggs originating from different females (egg clutches). First, data distribution was evaluated for normality using the Shapiro–Wilk test. Then, Pearson correlation coefficient was calculated for each gene expression in the dataset (in the gynogenetic (G) and control (C) groups separately) and survival rate in the corresponding egg clutches using JASP v.0.11.1 software (https://jasp-stats.org/). The genes with a statistically significant (with false discovery rate < 0.05 (FDR; by the Benjamini–Hochberg procedure)) correlation coefficient higher than 0.9 were further analyzed in detail for their function, using ShinyGO 0.76.3 software [25] (Ge et al., 2020). The genes were annotated in Gene Ontology (GO) biological processes (BP), molecular functions (MF), cellular components (CC) (http://geneontology.org/) and Kyoto Encyclopedia of Genes and Genomes (KEGG) databases.

The expression profiles of the mostly correlated genes were also used for the hierarchical clustering of the egg clutches and genes based on their expression profiles as well as for histogram plotting using ClustVis 2.0 software [26]. The expression levels of the separate genes were also compared between subgroups of females with low and high survival rates using Student’s *t* test in JASP software.

### 2.5. qPCR Validation of RNA-Seq Results

Six genes associated with the survival of embryos in the separate comparisons (Supplementary Files S2 and S3, Table S2) were selected for validation using alternative RT-qPCR method (*DAZ*-associated protein *2* gene (*dazap2*), follistatin-related protein *3* gene (*fstl3*), *PBX2* protein gene (*pbx2*), solute carrier family 9 member *A1b* gene (*slc9a1b*), *GTP*-binding protein *REM 2* gene (*rem2*), insulin receptor substrate 2a gene (*irs2a*). One of the genes (*dazap2*) was validated with the same RNA samples used for RNA-Seq. The remaining ones were validated based on new RNA isolates prepared from different eggs from the same batch of the same females. The primers were designed using https://primer3.ut.ee 4.1.0 tool, (accessed on 1 December 2024) and their sequences are shown in Supplementary File S6. Reverse Transcription PCR (RT-PCR) was performed using 0.2 μg of total RNA for each of the 14 samples (eggs from female no. 5 were eliminated from the analysis as described in the Material and Methods section) using a High-Capacity cDNA Reverse Transcription Kit (Thermo Fisher Scientific, Walthman, MA, USA) according to the standard protocol. Expression levels of the selected genes were quantified using the qPCR method in which a QuantStudio™ 3 Real-Time PCR System (Thermo Fisher Scientific, Walthman, MA, USA) was used with PowerUp™ SYBR™ Green Master Mix (Thermo Fisher Scientific, Walthman, MA, USA) as recommended by the manufacturer’s protocol. Target gene expression levels were normalized to ef1-a gene that was shown to be a good control for the rainbow trout eggs gene expression [27]. The gene expression levels were calculated by relative quantitative (RQ) analysis using the model proposed by Pfaffl [28], which takes into account a reaction efficiency for the individual genes. The data compliance among qPCR and RNA-Seq results was evaluated based on the correlation coefficient analysis using JASP 0.11.1 statistical software (https://jasp-stats.org/).

## 3. Results

### 3.1. Survival Rates of Embryos and Larvae

Eggs from eight females (F_1–8_) utilized in the current experiment did not display substantial inter-clutch variations in quality, as demonstrated by the comparable (*p* > 0.05) survival rates of their offspring from the control groups observed at the eyed stage as well as at the swim-up stage larvae (Table 1). The lowest survival of the rainbow trout embryos from the control groups was observed among progenies of female F_8_ (84.79 ± 4.63%), while decreased survival of swimming larvae was reported among progenies of female F_5_ (75.37 ± 18.69) (Table 1). In turn, the highest survival of the trout from the control groups was reported among embryos and larvae developing in eggs from female F_6_ (93.59 ± 3.79% and 93.29± 4.30%, respectively) (Table 1). On the other hand, the survival rates of individuals that hatched from the eggs activated by the UV-irradiated sperm and exposed to the HHP shock varied significantly (*p* < 0.05) from 3.04 ± 0.65 % (female F_2_) to 42.74 ± 2.2% (female F_7_) (eyed stage) (Table 1). Eggs stripped from females F_1_, F_4_, F_7_ and F_8_ exhibited several times higher competence for the gynogenetic development (expressed in the survival of the DHs) than eggs originated from females F_2_, F_3_, F_5_ and F_6_, irrespective of the stage of development (Table 1). Interestingly, the eggs originating from females F_3_, F_5_ and F_6_ showing a high survival rate after fertilization with non-irradiated milt did not exhibit any enhanced potential for gynogenetic development (Table 1). In turn, females F_1_, F_7_ and F_8_ produced eggs that showed decreased survival after fertilization with the non-irradiated trout semen but increased survival after gynogenetic activation (Table 1). Only eggs from female F_4_ had higher potential for normal and gynogenetic development, while only eggs from female F_2_ exhibited lower competence for development after activation with both irradiated and non-irradiated sperm (Table 1). In all females, the survival rates of offspring hatched from eggs activated with UV-irradiated semen were markedly lower (*p* < 0.05) when compared to the eggs inseminated with non-irradiated sperm (Table 1).

### 3.2. RNA-Seq Analysis

**Figure 1:**
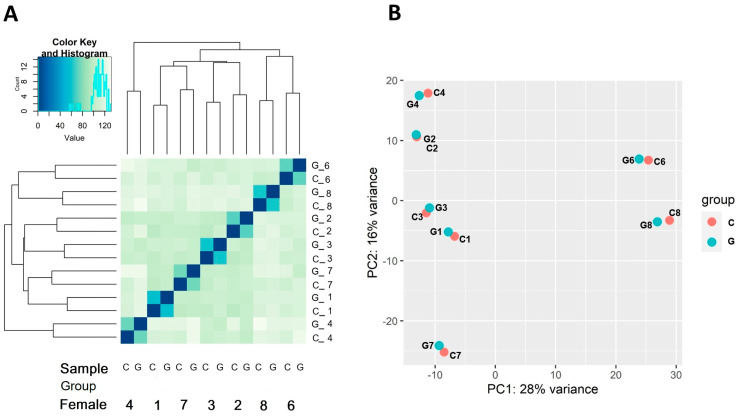
Clustering of expression profiles of the analyzed samples based on correlation coefficient (**A**) and principal components analysis (**B**). Clear clustering of samples according to females (1–8) but not treatment (G, C) can be observed.

In total, we generated RNA-Seq results for eggs from seven females utilized for the gynogenetic (G) and normal (C) activation. Sequencing produced on average 100 (group F) and 104 million (M) (group G) of raw PE reads per sample. After filtering, we used from 73 to 121 M of PE reads per sample for mapping, of which on average 73% were uniquely mapped against the newest available version of the genome. Of the mapped reads, a mean of 65% was mapped to annotation database (mainly genes) (Supplementary File S1). The applied annotation database (GTF) included Genscan gene predictions for 64,706 genes, of which 48,326 were coding genes and 15,627 were non-coding. The applied sequencing depth allowed to detect 30,919 genes with mean expression higher than one normalized read count. The global genes expression pattern varied between females (Figure 1).

### 3.3. Detecting Genes Associated with Survival Rate of Gynogenotes

**Figure 2:**
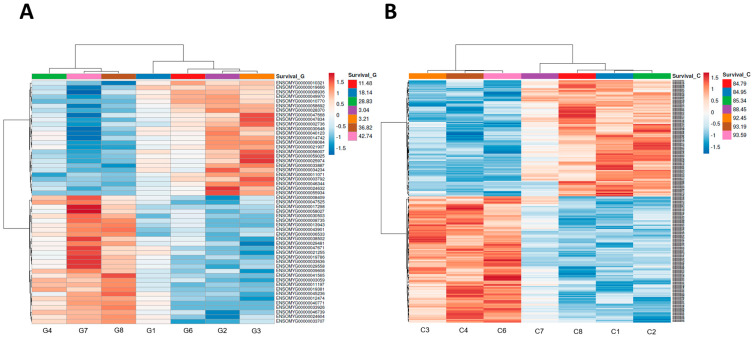
Hierarchical clustering of samples and expression profiles of genes mostly correlated with survival rates of rainbow trout from gynogenetic (**A**) and control (**B**) groups. Two major clusters of samples are visible, corresponding to eggs with high (G4, G7, G8) and low (G1, G2, G3, G6) survival rates after activation with UV-irradiated sperm (**A**) and high (C3, C4, C6) and low (C1, C2, C7, C8) survival rates after activation with non-irradiated sperm (**B**). Gene profiles are clustered into two main groups, corresponding to positive and negative correlations with the survival rates.

All expressed genes were further used for correlation analysis with the survival rates of rainbow trout developing in eggs originated from different females within the gynogenetic and control groups. Following data distribution analysis with the Shapiro–Wilk test, a Pearson correlation coefficient was applied. As the most relevant for survival rates, we selected genes with absolute value of correlation coefficient > 0.9 and statistically significant (FDR < 0.05). This approach allowed to detect 53 genes associated with the survival rates in the gynogenetic groups (G), and 221 in the control groups (C). None of the genes was common for G and C groups. In both G and C groups, the genes showed a nearly equal amount of positive and negative correlations. The list of genes, their expression values and correlation analyses along with their annotation are presented in Supplementary Files S2 and S3 for groups G and C, respectively. Hierarchical clustering applied based on the genes expression profiles allowed clear separation of eggs/females into two groups (clusters) with high and low survival rates (Figure 2). Eggs with similar transcriptomic profiles and high developmental competence for gynogenesis (survival rates above 28.9%) were stripped from females F_4_, F_7_ and F_8_ (Figure 2A), while eggs characterized by the high quality (survival after fertilization with non-irradiated sperm) that was above 92% and characterized by similar transcriptomic profiles originated from females F_3_, F_4_ and F_6_ (Figure 2B).

Transcripts associated with egg survival were verified with the RT-qPCR method. For *DAZAP2* gene, which was analyzed with qPCR based on the same RNA isolates used for the RNA-Seq, the compatibility between methods was moderate, as shown by a correlation coefficient of 0.464. For the *FSTL3* gene, for which other isolates of RNA were prepared from different eggs, this coefficient was lower and equal to 0.259 (*p* = 0.371). For the remaining genes that were analyzed only within the groups in which they were significant in RNA-Seq (7 samples), the correlation coefficient ranged from 0.16 (*PBX2*) to 0.76 (*IRS2a*) (Supplementary File S6). The moderate to low correlation of RNA-Seq and qPCR results found in this study probably results from the still not fully complete annotation of the rainbow trout genome that hampers the design of primers that are able to amplify all splicing variants of the validated genes.

The genes whose expression highly correlated with the survival rates of the rainbow trout were also analyzed for their functions. The classification of genes found in G group into GO BP categories showed that their high number was associated with developmental processes and cell differentiation, but they did not significantly enrich any of the GO annotation categories (Supplementary File S4). In the case of the genes found in the C group, they were mostly engaged in small molecule metabolic processes and microtubule-based processes and enriched (*p* < 0.05) only one GO category (CC) connected with CCAAT-binding factor complex (Supplementary File S5).

In Table 2, we present twenty fairly annotated and protein-coding genes most strongly associated with the survival rates of the gynogenetic embryos. The genes included interesting candidates for the development of eggs after gynogenetic activation, e.g., prolactin-releasing peptide receptor or follistatin-related protein 3. The expression of the most functionally interesting genes was analyzed in detail by comparing expression levels between females with high and low survival rates and analysis of expression profile variation.

## 4. Discussion

The production of diploid homozygous fish (DHs) may be inefficient due to the low survival rates of mitotic gynogenotes and androgenotes observed in many species [11]. The mortality of DHs is a complex issue primarily resulting from the expression of lethal alleles and the side effects of the physical shock applied during the duplication of uniparental chromosomes. Variations in the survival rates of gynogenotes developing from eggs sourced from different females have been observed in several fish species [29,30,31,32]. This indicates that the quality and origin of eggs (maternal effects) should also be considered as contributing factors that may partially explain the low yields of gynogenetic DHs. The quality of fish eggs is influenced by the rearing conditions, including environmental factors, husbandry practices, feeding, stress from handling, and overall welfare during oogenesis (1), the genetic status of the gamete donors (specifically their level of heterozygosity) (2), and the genetic and epigenetic regulation of maternal gene expression (3) [33,34]. In our research, all egg donors originated from the same broodstock and were reared under identical conditions, resulting in high and comparable egg quality. This quality was reflected by the similar survival rates of individuals that hatched from eggs fertilized with non-irradiated sperm (Table 1). Surprisingly, the developmental potential of eggs from different females varied significantly when activated by UV-irradiated spermatozoa and subjected to high hydrostatic pressure (HHP) shock (Table 1). Even more intriguing is the observation that eggs with the highest survival rates after fertilization with non-irradiated spermatozoa did not exhibit increased competence for gynogenetic development (Table 1). Several reports indicated that egg quality may affect efficiency of gynogenesis and androgenesis (reviewed by Ocalewicz [35]). However, eggs originating from different females may differ in the predisposition for gynogenesis, and this characteristic is not always related to egg quality sensu stricto (developmental competence). A female effect on the gynogenesis was previously proposed in the goldfish and crucian carp (*Carassius auratus* Linnaeus 1758), where eggs from different females exhibited different sensitivity to physical shocks applied for gynogenesis [36]. The exposure of gynogenetically activated eggs to sub-lethal physical treatment is an indispensable step for production of viable gynogenotes, since haploid fish suffer from so-called haploid syndrome and die during embryogenesis or shortly after hatching [37]. Paradoxically, the same physical treatment that recovers diploidy and enables generation of viable DH individuals is responsible for the decreased survival of gynogenetic diploid embryos; during the early stage of development, haploids show better survival than DHs [38]. Theoretically, the problem with egg oversensitivity to HHP shock might be overcome by using diploid eggs generated by tetraploid females for gynogenetic purposes. However, gynogenetic offspring provided by the activation of diploid eggs with UV-irradiated spermatozoa would not be fully homozygous. Moreover, low survival of tetraploids up to the maturation stage limits production of the diploid gynogenotes without application of physical shock.

Application of physical shock to eggs activated by irradiated spermatozoa at the prophase of the first mitosis in zygotes results in duplication of the maternal haploid set of chromosomes and thereby duplication of all alleles (also those that are deleterious). Expression of lethal alleles is considered the main factor responsible for the low survival of DHs. Nevertheless, such recessive alleles only act after the maternal to zygotic transition that occurs at the mid-blastula transition. In rainbow trout eggs incubated at 10 °C, mid-blastula transition takes place between the second and the third day after fertilization [39]. Prior zygotic genome activation, fish embryonic development is under control of the maternal mRNA deposited in the oocytes during oogenesis [40]. Therefore, early embryo mortality of DHs might be associated with expression of the maternal genome. The pattern of global gene expression in the rainbow trout eggs varied between clutches (females) (Figure 1), in accordance with our previous observations [41]. In spite of low differences between survival rates of progenies of all females from the control groups whose eggs were used in the experiment, about 200 genes whose expression was linked to survivability were found. This shows how much room there is to look for potential molecular markers of fish egg quality in salmonids.

Analysis of the maternal RNA showed that eggs with increased ability to develop after activation with UV-irradiated spermatozoa had different transcriptomic profiles of the maternal RNA than eggs with low competence for gynogenesis (Figure 2). Among twenty genes whose expression was substantially upregulated are those like *CASP 6* and *PGAM5* that are encoding proteins (caspase 6 and mitochondrial Serine/threonine-protein phosphatase, respectively) involved in the apoptotic processes [42]. Apoptosis facilitates elimination of damaged cells; therefore, it is an essential cellular mechanism during embryonic development [43]. This may be crucial in the case of induced gynogenesis when eggs are activated with UV-irradiated sperm cells and subjected to physical shock for ploidy elevation. An improper (too little) dose of radiation applied to the milt results in the incomplete inactivation of paternal chromosomes [44]. Radiation-induced chromosome fragments that are transferred to the zygote may impair further cell cleavages, destabilize genome organization, and finally, decrease survival of the DHs. Embryonic development of DHs may be also impaired by ploidy disturbances, including haploid/diploid mosaicism, observed sometimes in the gynogenetic specimens [38,45]. *PGAM5* protein has also been found to play an important role in the regulation of mitochondrial number and morphology [46,47]. Mitochondria and mitochondrial DNA influence oocyte quality, and the high number of mitochondria is essential for successful early embryogenesis [48,49]. Maternally inherited mitochondria supply the energy necessary for oocyte viability and early development until embryonic mitochondria take over their functions [50,51].

Gene-encoding GATA-binding protein 2a is another gene with increased expression observed in trout eggs exhibiting high potential for gynogenesis. GATA-binding proteins are a family of the transcription factors. Maternally expressed *GATA* may play an important role in the earliest transcription of the zygotic genome in the ascidians [52]. *GATA2*- and *GATA3*-encoding proteins are considered to be involved in early embryonic patterning in *Xenopus* and zebrafish (*Danio rerio*) [53,54,55]. Increased survival of rainbow trout gynogenetic DHs is also correlated with up-regulation of the transcription of gene encoding ras-like protein family member 11B (RASL11B). Proteins from the ras family are involved in the oocyte maturation and the prophase arrest in *Xenopus* [56,57]. The strict control of oocyte development and meiosis matters when further treatment after fertilization/egg activation needs to be applied at a particular moment, like the physical shock used for chromosome duplication during gynogenesis.

On the other hand, eggs that assure efficient development of gynogenetic DH rainbow trout also exhibit decreased expression levels of several genes whose function is to control: apoptosis (*Rem 2 GTPase*) follicular development (*FSTL3*), cell progression and divisions, RNA splicing, and protein synthesis (*phosphatase 1A-like*) [58,59,60]. Down regulation of some genes that are potentially associated with crucial embryonic development processes may suggest that their decreased expression is fair enough for the early cell cleavages.

Genes whose expression profiles were altered in eggs with different qualities and those showing increased potential for gynogenesis should be further extensively studied to find out their precise function in the rainbow trout and to provide molecular markers for trout egg quality and developmental competence for gynogenesis. Apart from genetic regulation, an analysis of the epigenetic changes in the DNA of rainbow trout eggs and gynogenetic embryos needs to also be considered, as such regulation of the gene expression was observed in gynogenetic cyprinids [61].

## Figures and Tables

**Table 1:** Survival rates (% ± SD) of gynogenetic and normal (parentheses) rainbow trout offspring developing in eggs originating from eight females. Different letters indicate significant differences (*p* < 0.05) in survival of gynogenetic embryos and larvae that hatched from eggs from different females.

Developmental Competence for Gynogenesis	Female	EYED STAGE	Swim-Up Stage
Increased	F_1_	18.14 ± 2.2 ^a^ (84.95 ± 4.97)	14.39 ± 2.49 ^a^ (80.01 ± 4.19)
	F_4_	28.83 ± 2.1 ^c^ (93.19 ± 2.31)	12.65 ± 0.43 ^c^ (88.81 ± 2.46)
	F_7_	42.74 ± 1.27 ^e^ (88.45 ± 0.78)	36.88 ± 1.70 ^e^ (82.82 ± 1.61)
	F_8_	36.82 ± 2.5 ^f^ (84.79 ± 4.63)	19.64 ± 4.49 ^f^ (79.71 ± 4.34)
Decreased	F_2_	3.04 ± 0.65 ^b^ (85.34 ± 2.90)	1.34 ± 0.25 ^b^ (77.89 ± 4.66)
	F_3_	3.21 ± 0.33 ^b^ (92.45 ± 1.70)	2.54 ± 0.36 ^b^ (92.12 ± 1.64)
	F_5_	5.93 ± 1.47 ^b^ (90.13 ± 3.52)	1.93 ± 0.43 ^b^ (75.37 ± 18.69)
	F_6_	11.48 ± 1.47 ^d^ (93.59 ± 3.79)	6.13 ± 1.0 ^d^ (93.29 ± 4.30)

**Table 2:** Protein coding and well-annotated genes showing the strongest correlation of expression profile with the survival of gynogenetically activated eggs.

ENSEMBL ID	Gene Symbol	Correlation Coefficient	Gene Name
ENSOMYG00000028370	*REM2*	−0.982	GTP-binding protein REM 2-like
ENSOMYG00000012474		0.973	interferon regulatory factor 4-like
ENSOMYG00000030648	*LRRC47*	−0.967	leucine rich repeat containing 47
ENSOMYG00000008084	*HESX1*	−0.962	HESX homeobox 1
ENSOMYG00000040771	*IRS2a*	0.962	insulin receptor substrate 2
ENSOMYG00000033636	*TMEM97*	0.96	transmembrane protein 97
ENSOMYG00000038502	*CASP6*	0.954	caspase 6
ENSOMYG00000008735		0.952	T-cell ecto-ADP-ribosyltransferase 1-like
ENSOMYG00000021907	*MYRIP*	−0.949	Danio rerio myosin VIIA and Rab interacting protein (myrip)
ENSOMYG00000046739	*PRLHR2A*	0.948	prolactin-releasing peptide receptor-like
ENSOMYG00000047671	*PGAM5*	0.946	serine/threonine-protein phosphatase PGAM5, mitochondrial-like
ENSOMYG00000013943	*PPDPFA*	0.944	pancreatic progenitor cell differentiation and proliferation factor-like
ENSOMYG00000041565	*GATA2*	0.943	GATA binding protein 2a
ENSOMYG00000011197	*OXSR1*	0.942	oxidative stress responsive kinase 1a
ENSOMYG00000019666	*FSTL3*	−0.94	follistatin-related protein 3-like
ENSOMYG00000047834		−0.939	Oncorhynchus mykiss Sec61 alpha form B (LOC100135961)
ENSOMYG00000019786	*ALX*	0.936	ALX homeobox protein 1-like
ENSOMYG00000010770	*PPM1AB*	−0.936	protein phosphatase 1A-like
ENSOMYG00000003059	*RASl11B*	0.929	ras-like protein family member 11B
ENSOMYG00000029481	*FBXW11A*	0.924	F-box/WD repeat-containing protein 11

## Data Availability

Data is available at the following links: https://www.ncbi.nlm.nih.gov/geo/query/acc.cgi?acc=GSE225353 (accessed on 3 July 2025), https://www.ncbi.nlm.nih.gov/Traces/study/?acc=PRJNA935331&o=acc_s%3Aa (accessed on 3 July 2025), https://www.ncbi.nlm.nih.gov/bioproject/PRJNA935331 (accessed on 3 July 2025).

## Supplementary Materials

The following supporting information can be downloaded at https://www.mdpi.com/article/10.3390/genes16070803/s1, Table S1: Length and weight of the gamete donors for the expriment, Files S1–S6: Database.

## References

[1] Lowe R., Shirley N., Bleackley M., Dolan S., Shafee T. (2017). Transcriptomics technologies. PLoS Comput. Biol..

[2] Mutz K.O., Heilkenbrinker A., Lönne M., Walter J.G., Stahl F. (2013). Transcriptome analysis using next-generation sequencing. Curr. Opin. Biotechnol..

[3] Chandhini S., Rejish Kumar V.J. (2019). Transcriptomics in aquaculture: Current status and applications. Rev. Aqua..

[4] Bonnet E., Fostier A., Bobe J. (2007). Microarray-based analysis of fish egg quality after natural or controlled ovulation. BMC Genomics.

[5] Lanes C.F.C., Bizuayehu T.T., de Oliveira Fernandes J.M., Kiron V., Babiak I. (2013). Transcriptome of Atlantic cod (*Gadus morhua* L.) early embryos from farmed and wild broodstocks. Mar. Biotechnol..

[6] Aegerter S., Jalabert B., Bobe J. (2005). Large scale real-time PCR analysis of mRNA abundance in rainbow trout eggs in relationship with egg quality and post-ovulatory ageing. Mol. Reprod. Dev..

[7] Chapman R.W., Reading B.J., Sullivan C.V. (2014). Ovary transcriptome profiling via artificial intelligence reveals a transcriptomic fingerprint predicting egg quality in striped bass, *Morone saxatilis*. PLoS ONE.

[8] Lanes C.F., Fernandes J.M., Kiron V., Babiak I. (2012). Profiling of key apoptotic, stress, and immune-related transcripts during embryonic and postembryonic development of Atlantic cod (*Gadus morhua* L.). Theriogenology.

[9] Mommens M., Fernandes J.M., Bizuayehu T.T., Bolla S.L., Johnston I.A., Babiak I. (2010). Maternal gene expression in Atlantic halibut (*Hippoglossus hippoglossus* L.) and its relation to egg quality. BMC Res. Notes.

[10] Pandian T.J., Koteeswaran R. (1998). Ploidy induction and sex control in fish. Hydrobiologia.

[11] Komen H., Thorgaard G., A (2007). Androgenesis, gynogenesis and the production of clones in fishes: A review. Aquaculture.

[12] Franek R., Baloch A.R., Kaspar V., Saito T., Fujimoto T., Arai K., Psenicka M. (2020). Isogenic lines in fish—A critical review. Rev. Aquac..

[13] Liu S., Zhang Y., Zhou Z., Waldbieser G., Sun F., Lu J., Zhang J., Jiang Y., Zhang H., Wang X. (2012). Efficient assembly and annotation of the transcriptome of catfish by RNA-Seq analysis of a doubled haploid homozygote. BMC Genomics.

[14] Jagiełło K., Dobosz S., Zalewski T., Polonis M., Ocalewicz K. (2018). Developmental competence of eggs produced by rainbow trout Doubled Haploids (DHs) and generation of the clonal lines. Reprod. Domest. Anim..

[15] Ocalewicz K., Gurgul A., Polonis M., Dobosz S. (2020). Preliminary identification of candidate genes related to survival of gynogenetic rainbow trout (*Oncorhynchus mykiss*) based on comparative transcriptome analysis. Animals.

[16] Rożyński R., Kuciński M., Dobosz S., Ocalewicz K. (2023). Successful application of UV-irradiated rainbow trout (*Oncorhynchus mykiss*) spermatozoa to induce gynogenetic development of the European grayling (*Thymallus thymallus*). Aquaculture.

[17] Billard R. (1992). Reproduction in rainbow trout: Sex differentiation, dynamics of gametogenesis, biology and preservation of gametes. Aquaculture.

[18] Polonis M., Fujimoto T., Dobosz S., Zalewski T., Ocalewicz K. (2018). Genome incompatibility between rainbow trout (*Oncorhynchus mykiss*) and sea trout (*Salmo trutta*) and induction of the interspecies gynogenesis. J. Appl. Genet..

[19] Ocalewicz K., Gurgul A., Pawlina-Tyszko K., Szmatoła T., Jasielczuk I., Bugno-Poniewierska M., Dobosz S. (2019). Induced androgenetic development in rainbow trout and transcriptome analysis of irradiated eggs. Sci. Rep..

[20] Wingett S.W., Andrews S. (2018). FastQ Screen: A tool for multi-genome mapping and quality control. F1000Research.

[21] Dodt M., Roehr J.T., Ahmed R., Dieterich C. (2012). FLEXBAR-Flexible Barcode and Adapter Processing for Next-Generation Sequencing Platforms. Biology.

[22] Dobin A., Davis C.A., Schlesinger F., Drenkow J., Zaleski C., Jha S., Batut P., Chaisson M., Gingeras T.R. (2013). STAR: Ultrafast universal RNA-seq aligner. Bioinformatics.

[23] Anders S., Pyl P.T., Huber W. (2015). HTSeq–a Python framework to work with high-throughput sequencing data. Bioinformatics.

[24] Love M.I., Huber W., Anders S. (2014). Moderated estimation of fold change and dispersion for RNA-seq data with DESeq2. Genome. Biol..

[25] Ge S.X., Jung D., Yao R. (2020). ShinyGO: A graphical gene-set enrichment tool for animals and plants. Bioinformatics.

[26] Metsalu T., Vilo J. (2015). ClustVis: A web tool for visualizing clustering of multivariate data using Principal Component Analysis and heatmap. Nucleic Acids Res..

[27] Bland F., McIntosh R., Bain N., Snow M. (2012). Development and validation of a range of endogenous controls to support the implementation of practical Taqman real-time PCR-based surveillance for fish diseases within aquaculture. J. Fish. Dis..

[28] Pfaffl M.W. (2001). A new mathematical model for relative quantification in real-time RT-PCR. Nucleic Acids Res..

[29] Naruse K., Ijiri K., Shima A., Egami N. (1985). The production of cloned fish in the Medaka (*Oryzias latipes*). J. Exp. Zool..

[30] Komen J., Bongers A.B.J., Richter C.J.J., van Muiswinkel W.B., Huisman E.A. (1991). Gynogenesis in common carp (*Cyprinus carpio* L.) II. The production of homozygous gynogenetic clones and F_1_ hybrids. Aquaculture.

[31] Arai K. (2001). Genetic improvement of aquaculture finfish species by chromosome manipulation techniques in Japan. Aquaculture.

[32] Quillet E., Garcia P., Guyomard R. (1991). Analysis of the production of all homozygous lines of rainbow trout by gynogenesis. J. Exp. Biol..

[33] Migaud H., Bell G., Cabrita E., McAndrew B., Davie A., Bobe J., Herraez M.P., Carrillo M. (2013). Gamete quality and broodstock management in temperate fish. Rev. Aquaculture.

[34] Bobe J. (2015). Egg quality in fish: Present and future challenges. Anim. Front..

[35] Ocalewicz K. (2024). Quality of fish eggs and production of androgenetic and gynogenetic doubled haploids (DHs). Fish. Physiol. Biochem..

[36] Yamaha E., Otani S., Minami A., Arai K. (2002). Dorso-ventral axis perturbation in goldfish embryos caused by heat- and pressure-shock treatments for chromosomes set manipulation. Fish. Sci..

[37] Luo C., Li B. (2003). Diploid-dependent regulation of gene expression: A genetic cause of abnormal development in fish haploid embryos. Heredity.

[38] Michalik O., Dobosz S., Zalewski T., Sapota M., Ocalewicz K. (2015). Induction of gynogenetic and androgenetic haploid and doubled haploid development in the brown trout (*Salmo trutta Linnaeus* 1758). Reprod. Domest. Anim..

[39] Takeuchi Y., Yoshizaki G., Takeuchi T. (1999). Green fluorescent protein as a cell-labelling tool and a reporter of gene expression in transgenic rainbow trout. Marine Biotech..

[40] Pelegri F. (2003). Maternal factors in zebrafish development. Dev. Dyn..

[41] Gurgul A., Pawlina-Tyszko K., Bugno-Poniewierska M., Szmatoła T., Jasielczuk I., Dobosz S., Ocalewicz K. (2018). Transcriptome analysis of rainbow trout (*Oncorhynchus mykiss*) eggs subjected to the high hydrostatic pressure treatment. Int. J. Genomics..

[42] Shalini S., Dorstyn L., Dawar S., Kumar S. (2015). Old, new and emerging functions of caspases. Cell Death Differ..

[43] Elmore S. (2007). Apoptosis: A review of programmed cell death. Toxicol. Pathol..

[44] Krisfalusi M., Wheeler P.A., Thorgaard G.H., Cloud J.G. (2000). Gonadal morphology of female diploid gynogenetic and triploid rainbow trout. J. Exp. Zool..

[45] Fopp-Bayat D., Ocalewicz K., Kucinski M., Jankun M., Laczynska B. (2017). Disturbances in the ploidy level in the gynogenetic sterlet *Acipenser ruthenus*. J. Appl. Genet..

[46] Cheng M., Lin N., Dong D., Ma J., Su J., Sun L. (2021). PGAM5: A crucial role in mitochondrial dynamics and programmed cell death. Eur. J. Cell Biol..

[47] Nag S., Szederkenyi K., Gorbenko O., Tyrrell H., Yip C.M., McQuibban G.A. (2023). PGAM5 is an MFN2 phosphatase that plays an essential role in the regulation of mitochondrial dynamics. Cell Rep..

[48] Wang L.Y., Wang D.H., Zou X.Y., Xu C.M. (2009). Mitochondrial functions on oocytes and preimplantation embryos. J. Zhejiang Univ. Sci. B..

[49] Chappel S. (2013). The role of mitochondria from mature oocyte to viable blastocyst. Obstet. Gynecol. Int..

[50] Wilding M., Coppola G., Dale B., Di Matteo L. (2009). Mitochondria and human preimplantation embryo development. Reproduction..

[51] Artuso L., Romano A., Verri T., Domenichini A., Argenton F., Santorelli F.M., Petruzzella V. (2012). Mitochondrial DNA metabolism in early development of zebrafish (*Danio rerio*). Biochim. Biophys. Acta.

[52] Imai K.S., Kobayashi K., Kari W., Rothbächer U., Ookubo N., Oda-Ishii I., Satou Y. (2020). Gata is ubiquitously required for the earliest zygotic gene transcription in the ascidian embryo. Dev. Biol..

[53] Zon L.I., Mather C., Burgess S., Bolce M.E., Harland R.M., Orkin S.H. (1991). Expression of GATA-binding proteins during embryonic development in Xenopus laevis. Proc. Natl. Acad. Sci. USA.

[54] Neave B., Rodaway A., Wilson S.W., Patient R., Holder N. (1995). Expression of zebrafish GATA 3 (gta3) during gastrulation and neurulation suggests a role in the specification of cell fate. Mech. Dev..

[55] Sykes T.G., Rodaway A.R., Walmsley M.E., Patient R.K. (1998). Suppression of GATA factor activity causes axis duplication in *Xenopus*. Development..

[56] Birchmeier C., Broek D., Wigler M. (1985). Ras proteins can induce meiosis in Xenopus oocytes. Cell.

[57] Jessus C., Rime H., Ozon R. (1998). Ras family proteins: New players involved in the diplotene arrest of *Xenopus oocytes*. Biol Cell..

[58] Edel M.J., Boué S., Menchon C., Sánchez-Danés A., Izpisua Belmonte J.C. (2010). Rem2 GTPase controls proliferation and apoptosis of neurons during embryo development. Cell Cycle.

[59] He J., Liu Q., Yu S., Lei M., Liu J., Di R., Ge Z., Hu W., Wang X., Liu N. (2021). Expression and functional analysis of the Follistatin-like 3 (FSTL3) gene in the sheep ovary during the oestrous cycle. Reprod. Domest. Anim..

[60] Xia Y., Sidis Y., Schneyer A. (2004). Overexpression of follistatin-like 3 in gonads causes defects in gonadal development and function in transgenic mice. Mol. Endocrinol..

[61] Qin Q., Wang C., Zhou Y., Qin H., Zhao C., Yang L., Yu T., Liu S. (2020). Rapid Genomic and Epigenetic Alterations in Gynogenetic Carassius auratus Red Var. Derived from Distant Hybridization. Mar. Biotechnol..

