# Supplementary material for: Transcriptome Profiling Reveals Differences Between Rainbow Trout Eggs with High and Low Potential for Gynogenesis
Source: Genes (Basel). 2025 Jul 8;16(7):803. doi: 10.3390/genes16070803 (PMC12294859; doi:10.3390/genes16070803)
Supplement: Supplementary file 1 [file genes-16-00803-s001.zip › Supplementary File 1.pdf]

Sample	Female	Group	Number of raw reads (pairs)	Number of filtered reads (pairs)	Percent of uniquely mapped reads (pairs)	Number of uniquely mapped reads (pairs)	Percent of reads mapped to annotation database
9	1	Control (C)	99,657,000	97,702,942	79.2	77,332,208	65
10	2		112,547,760	110,340,942	70.2	77,459,900	64
11	3		104,523,698	102,474,214	75.4	77,305,574	67
12	4		121,102,429	118,727,872	70.6	83,803,860	69
14	6		97,951,148	96,030,538	67.2	64,557,452	69
15	7		75,376,790	73,898,814	67.8	50,065,096	61
16	8		91,910,496	90,108,330	76.1	68,571,012	65
Group (mean)		C	100,438,474	98,469,093	72.4	71,299,300	66
17	1	Gynogenesis (G)	96,656,448	94,761,224	80.0	75,838,302	64
18	2		103,700,450	101,667,108	64.0	65,014,334	67
19	3		104,399,354	102,352,308	73.2	74,895,816	64
20	4		109,164,233	107,023,758	63.8	68,230,892	69
22	6		113,649,950	111,421,520	78.6	87,596,166	61
23	7		113,056,208	110,839,420	81.8	90,664,670	65
24	8		123,644,975	121,220,564	69.8	84,653,880	64
Group (mean)		G	109,181,660	107,040,843	73.0	78,127,723	65
All (mean)		Both	104,810,067	102,754,968	73	74,713,512	65
