# Supplementary material for: Transcriptome Profiling Reveals Differences Between Rainbow Trout Eggs with High and Low Potential for Gynogenesis
Source: Genes (Basel). 2025 Jul 8;16(7):803. doi: 10.3390/genes16070803 (PMC12294859; doi:10.3390/genes16070803)
Supplement: Supplementary file 1 [file genes-16-00803-s001.zip › Supplementary Table 1.pdf]

Supplementary Table S1. Length and weight of the gamete donors for the experiment

Gamete donors		Total length (mm)	Weight (g)
females	males		
F_1		540	2440
F_2		615	3424
F_3		565	2798
F_4		655	4052
F_5		560	3242
F_6		550	3198
F_7		630	3804
F_8		560	2606
	rainbow trout 1	540	2288
	grayling 1	400	552
	grayling 2	325	266
	grayling 3	310	242
